# Weight loss, dysphagia and supplement intake in patients with amyotrophic lateral sclerosis (ALS): impact on quality of life and therapeutic options

**DOI:** 10.1186/1471-2377-13-84

**Published:** 2013-07-12

**Authors:** Sonja Körner, Melanie Hendricks, Katja Kollewe, Antonia Zapf, Reinhard Dengler, Vincenzo Silani, Susanne Petri

**Affiliations:** 1 Department of Neurology, Hannover Medical School, Carl-Neuberg-Str. 1, Hannover 30625, Germany; 2 Center for Systems Neuroscience (ZSN), Hannover, Germany; 3 Department of Medical Statistics, University Göttingen, Göttingen, Germany; 4 Department of Neurology and Laboratory of Neuroscience, IRCCS Istituto Auxologico Italiano, Milan 20149, Italy; 5 Department of Pathophysiology and Transplantation, “Dino Ferrari” Center, Università degli Studi di Milano, Milan 20149, Italy

**Keywords:** Amyotrophic lateral sclerosis, Nutrition, Dietary supplements, Weight loss, Dysphagia, High calorie supplements

## Abstract

**Background:**

Weight loss is a frequent feature in the motor neuron disease Amyotrophic lateral sclerosis (ALS). In this study we investigated possible causes of weight loss in ALS, its impact on mood/quality of life (QOL) and the benefit of high calorie nutritional/other dietary supplements and percutaneous endoscopic gastrostomy (PEG).

**Methods:**

121 ALS patients were interviewed and answered standardized questionnaires (Beck depression inventory - II, SF36 Health Survey questionnaire, revised ALS functional rating scale). Two years after the initial survey we performed a follow-up interview.

**Results:**

In our ALS-cohort, 56.3% of the patients suffered from weight loss. Weight loss had a negative impact on QOL and was associated with a shorter survival. Patients who took high calorie nutritional supplements respectively had a PEG stated a great benefit regarding weight stabilization and/or QOL.

38.2% of our patients had significant weight loss without suffering from dysphagia. To clarify the reasons for weight loss in these patients, we compared them with patients without weight loss. The two groups did not differ regarding severity of disease, depression, frontotemporal dementia or fasciculations, but patients with weight loss declared more often increased respiratory work.

**Conclusions:**

Weight loss is a serious issue in ALS and cannot always be attributed to dysphagia. Symptomatic treatment of weight loss (high calorie nutritional supplements and/ or PEG) should be offered more frequently.

## Background

Amyotrophic lateral sclerosis (ALS) is the most common adult-onset neurodegenerative disorder of the motor system. It is characterized by loss of upper and lower motor neurons in the primary motor cortex, brainstem and spinal cord. The resulting paralyses are rapidly progressive and lead to death due to respiratory failure within 2–5 years [1]. Weight loss is a frequent phenomenon in ALS. It occurs not only in association with dysphagia but also due to not yet fully understood disease-specific reasons. Hypotheses to explain weight loss in ALS include higher waste of energy because of muscle fasciculations, increasing respiratory efforts, hypermetabolism and decreased food intake due to depression [2-4]. In any case it is well known that weight loss and a lower body-mass-index (BMI) are negative prognostic factors for survival in ALS [5-8]. High-energy diet prolonged survival in transgenic ALS mice [9]. However, administration of high calorie nutritional supplements or percutaneous endoscopic gastrostomy (PEG) in case of weight loss is not considered early and frequently enough.

In contrast, self-medication with other dietary supplements, also called “nutriceuticals “ or “functional food” has become increasingly popular among ALS patients and according to the literature is used by up to 80% of them [7,10]. Dietary supplements are supposed to affect diverse mechanisms leading to motor neuron death or alleviate symptoms of ALS, often based on theoretical benefits or anecdotal reports [7]. Frequently they are self-prescribed and patients take several dietary supplements simultaneously.

The aim of the present study was to investigate the extent of weight loss in ALS and to analyse the impact of weight loss on mood, quality of life (QOL) and survival of ALS patients. Additionally potential underlying causes of weight loss beyond dysphagia should be evaluated. Further we were interested in the frequency and benefit of PEG insertion, high-calorie supplement (e.g. calorically dense shakes/drinks) and other dietary supplement (e.g. vitamins, homeopathic medication) intake in ALS-patients.

## Methods

121 ALS patients from the ALS outpatient clinic at Hannover Medical School participated in the survey. The study has been approved by the ethical committee of the Hannover Medical School and all subjects gave informed consent to the participation. All patients had met the revised El Escorial criteria for probable or definite ALS. Only in one patient, subtle clinical signs of frontotemporal dementia (FTD) were present. FTD was underrepresented in our population because these patients are less motivated and less suitable to participate in this type of study. Patients filled in three standardized questionnaires (Beck depression inventory - II (BDI), SF-36 Health Survey questionnaire (SF-36) and revised ALS functional rating scale (ALS-FRS-R)) and were further interviewed about weight loss, dysphagia, food habits and their intake of dietary supplements. It was documented if patients suffered fasciculations or respiratory distress (yes or no).

Two years after the initial survey, 61.2% of the patients or their relatives were available for a short follow-up telephone interview to find out whether the patient was still alive and whether the patient still used dietary supplements.

The ALSFRS_R is a well-established and widely used score for the functional status of patients with ALS [11]. It is based on 12 items, each rated on a 0–4 point scale. The rate of functional disability ranges from 0 (maximum disability) to 48 (normal) points. Three items of the ALSFRS_R assess bulbar involvement (speech, salivation, swallowing), which therefore can be rated from 0 (maximum bulbar involvement) to 12 (no bulbar involvement).

The BDI is a 21-question multiple-choice self-report inventory and a commonly used instrument for quantifying levels of depression. Each of the 21 items is scored on a scale value of 0 (symptom not present) to 3 (symptom very intense) leading to an overall-range of 63. The cutoffs used are 0–8: no depression, 9–13: minimal depression, 14–19: mild depression, 20–28: moderate depression, 29–63 severe depression [12].

The SF36 questionnaire is a multi-purpose, short-form health survey with 36 questions. It is a self-administered QOL scoring system that includes eight independent scales: 1. physical functioning (limitations in physical activities), 2. Physical role (limitations in usual role activities because of physical health problems), 3. Bodily pain, 4. General health perception, 5. Vitality (energy and fatigue), 6. social functioning (limitations in social activities because of physical or emotional problems), 7. Emotional role (limitations in usual role activities because of emotional problems), 8. Mental health (psychological distress and well-being) [13]. The SF-36 questionnaire is widely used and appropriate in ALS patients [14].

The tests and questionnaires used in this study are briefly summarized in Table 1.

**Table 1:** Applied tests/questionnaires and their range and meaning

**Test/questionnaires**	**Range**
Self designed questionnaire:	
• Collected information about presence of weight loss, dysphagia, supplement intake, fasciculations, respiratory effort, high calorie supplement intake, PEG	**•** patients had to choose “yes” or “no”
• Included the questions:	• patients had to choose one of the answers
- How did the PEG insertion/high calorie supplement intake affect your weight?	- “Further weight loss”, “weight stabilization”, “weight gain”
- How did the PEG insertion influence your quality of life?	- “strong improvement”, “moderate improvement”, “decline”, “no influence”
• Collected data about age, duration of disease, region of onset, sort of supplement.	• free text
ALSFRS_R total	**0** (maximum disability) to **48** (normal)
ALSFRS_R bulbar	**0** (maximum bulbar involvement) to **12** (no bulbar involvement)
BDI	**0** (no depression) to **63** (severe depression)
SF36	**0** (poorest health) to **100** (best health)

Patients were divided in the following groups: “weight loss” (defined as >3 kg since disease onset), again subdivided in “weight loss without/with dysphagia” and “no weight loss” and, for the second aspect of the study, “with supplement intake” and “without supplement intake” (supplement defined here as dietary supplements (e.g. vitamins)). The groups did not differ regarding gender or site of onset. Disease severity (ALSFRS_R), extent of depression (BDI) and quality of life (SF36) were compared between these patient groups (group composition see Table 2) using t-tests for independent samples. To identify correlations between weight loss or supplement intake and QOL independent from disease severity (ALSFRS-R) we performed multiple regression analysis (dependent variable: SF-36 subscale, predictor variables: ALSFRS-R and weight loss or supplement intake). Statistical analyses were performed using SPSS V. 19 (SPSS, Chicago, IL) software, a p-value of <0.05 was considered significant.

**Table 2:** Group composition and characteristics of patients with and without dietary supplement intake/weight loss

	**Number of patients**	**Gender**	**Mean age (years)**	**Mean disease duration (months)**	**Mean ALSFRS_R total**	**Mean ALSFRS_R bulbar**	**Percentage of bulbar onset patients (%)**
total	121	81 male 40 female	59.74	41.4	29.29	8.66	11.6
with supplement intake	67*	49 male 18 female	56.63	36.7	31.11	9.4	6.0
without supplement intake	50*	30 male 20 female	63.56	45.8	26.92	7.83	14.0
weight loss	68	47 male 21 female	61.0	30	27.5	7.67	14.7
weight loss with dysphagia	42	29 male 13 female	60.67	29	22.9	5.56	21.4
weight loss without dysphagia	26	18 male 8 female	61.54	31.8	30.52	11.12	3.8
no weight loss	53	34 male 19 female	58,11	54.5**	33.82	9.94	7.5

* Four patients did not make any statement regarding supplement intake.

** Disease duration significant different from the groups “weight loss” and “weight loss with dysphagia”.

## Results

**Figure 1:**
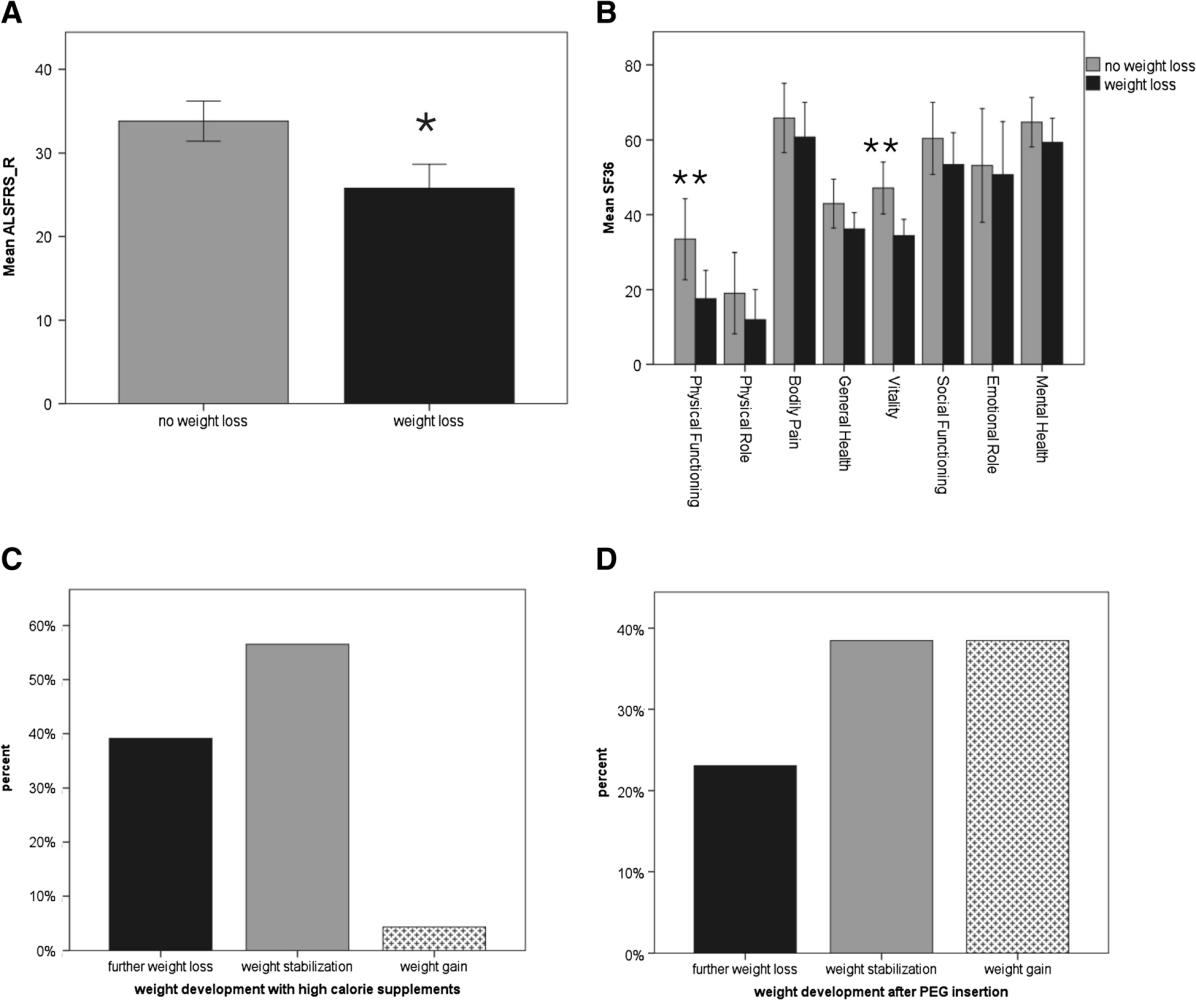
**Mean scores of ALSFRS_R and SF36 questionnaire of patients without and with weight loss and impact of high calorie supplements/PEG.** Mean scores of ALSFRS_R (**A**) and SF36 questionnaire (**B**) of patients without and with weight loss. The latter group showed significantly lower scores at the ALSFRS_R and the SF36 subscales “physical functioning” and “vitality”. 58.3% of patients consuming high calorie supplements and 76.9% of patients who had undergone PEG reported subsequent weight stabilization or even weight gain (**C** and **D**). * p < 0.05, ** p < 0.01.

56.2% (n = 68) of the patients in our cohort reported about weight loss. Weight loss was associated with a significantly worse ALSFRS_R score and also with higher depression (BDI, not significant) and significantly lower QOL scores (SF36) regarding the subscales “physical functioning” and “vitality” (Figure 1A and B). Multiple regression analysis identified the ALSFRS_R score as confounding factor, showing that the differences in BDI and “physical functioning” were probably caused by the discrepancy in the ALSFRS_R. But the difference in the SF36 subscale “vitality” between patients with and without weight loss remained highly significant (Table 3A), which means that patients with weight loss feel more often exhausted, tired and spiritless, regardless of the disease stage. Multiple regression analysis showed that this influence of weight loss on vitality was independent of respiratory distress which had a significant effect on vitality as well (Table 3B).

**Table 3:** Multiple regression analysis of the SF36 subscale “vitality” and weight loss (A and B) respectively the SF36 subscale “social functioning” and supplement intake (C)

		
A		
**Vitality**	**Regression coefficient**	**p-value**
(SF-36 scale)		
ALSFRS_R	0,416	**0,013**
Weight loss	−9,281	**0,011**
B		
**Vitality**	**Regression coefficient**	**p-value**
(SF-36 scale)		
Respiratory distress	−10.093	**0,005**
Weight loss	−9,457	**0,008**
C		
**Social Functioning**	**Regression coefficient**	**p-value**
(SF-36 scale)		
ALSFRS_R	0,628	**0,010**
Supplement intake	14,342	**0,008**

Multiple regression analysis of the SF36 subscale “vitality” and weight loss (A and B) respectively the SF36 subscale “social functioning” and supplement intake (C) taking into account the influence of the ALSFRS_R score/respiratory distress. The calculations showed an independent significant influence of both weight loss/supplement intake and ALSFRS_R score/respiratory distress on “vitality” respectively “social functioning”: Patients with weight loss and lower ALSFRS_R showed worse results regarding “vitality” (A and B) and patients with supplement intake and higher ALSFRS_R showed better results regarding “social functioning” (C).

33.8% (n = 23) of patients with weight loss consumed high calorie supplements and 60.8% (n = 14) of these reported subsequent weight stabilization or even weight gain (Figure 1C). 25.5% (n = 13) of patients with dysphagia had undergone PEG; 76.9% (n = 10) of these patients declared weight stabilization or weight gain (Figure 1D) and 84.6% (n = 11) stated an improvement of QOL after PEG insertion. Remarkably, no patient indicated deterioration of QOL after PEG insertion (although this is often suspected by patients and relatives prior to the procedure).

**Figure 2:**
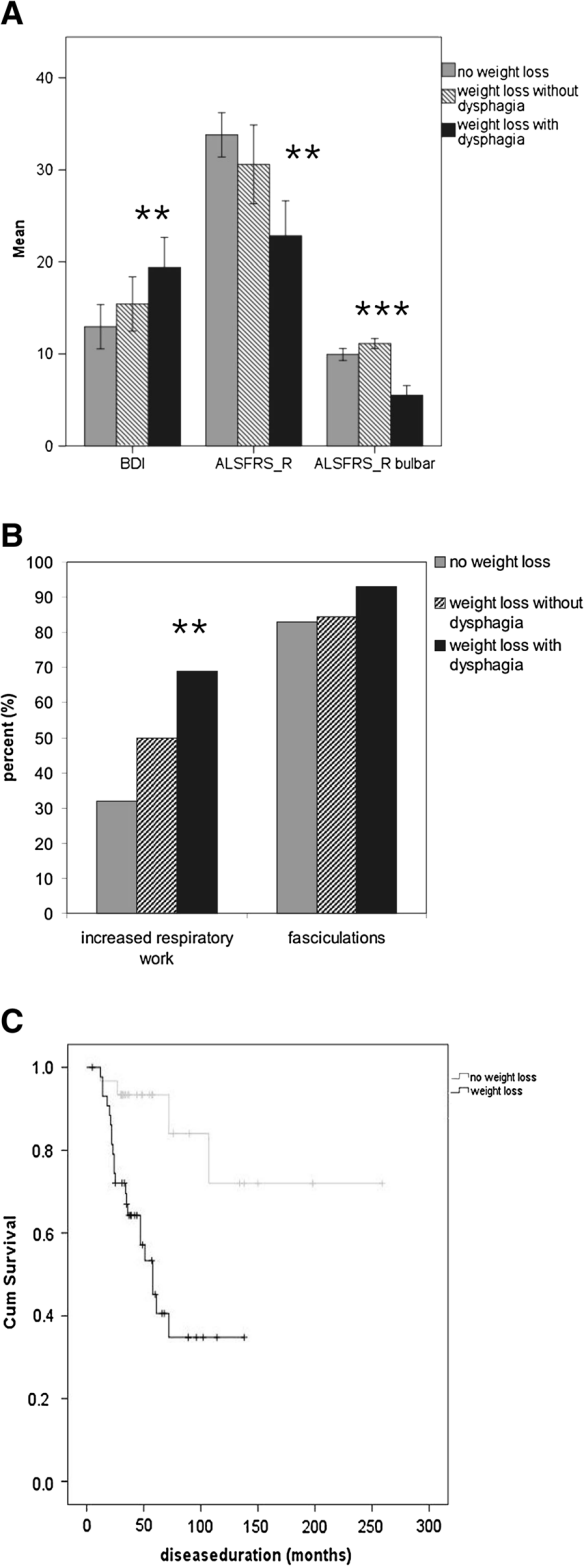
**Comparison of patients with weight loss with/without dysphagia and patients without weight loss.** Patients with weight loss and dysphagia differed significantly from patients without weight loss in BDI and ALSFRS_R (total and bulbar). Patients with weight loss without dysphagia, on the other hand, did not have higher BDI/ lower ALSFRS_R scores than patients without weight loss (**A**). Weight loss in patients without dysphagia therefore does not seem to be directly related to a more advanced disease stage or increased depression. Patients with weight loss and dysphagia significantly more often declared increased respiratory work than patients without weight loss. Patients with weight loss without dysphagia showed a tendency towards increased respiratory work compared to patients without weight loss (p = 0.12). There were no differences regarding frequency of fasciculations between the groups (**B**). Follow-up by telephone two years after the initial survey highlighted the prognostic value of weight loss: Kaplan-Meier survival analysis for ALS patients with and without weight loss revealed significantly shorter survival of ALS patients with weight loss (log rank p = 0.001) (**C**).

38.2% (n = 26) of patients with weight loss did not suffer from dysphagia (according to self-reported statement and ALSFRS_R). This patient group did not differ from patients without weight loss regarding ALSFRS_R (total and bulbar) and depression (BDI) nor did they report changes in their eating habits. Patients with dysphagia on the other hand showed significantly lower ALSFRS_R scores, mainly due to the bulbar subscore (Figure 2A). The prevalence of fasciculations in patients with weight loss without dysphagia was equal to patients without weight loss. They did, however, more often declare increased respiratory efforts compared to patients without weight loss (Figure 2B).

The telephone survey after two years showed that weight loss was a strong negative prognostic factor: Kaplan- Meier survival curves of patients with and without weight loss showed significantly shorter survival of patients with weight loss (log rank p = 0.001) (Figure 2C).

**Figure 3:**
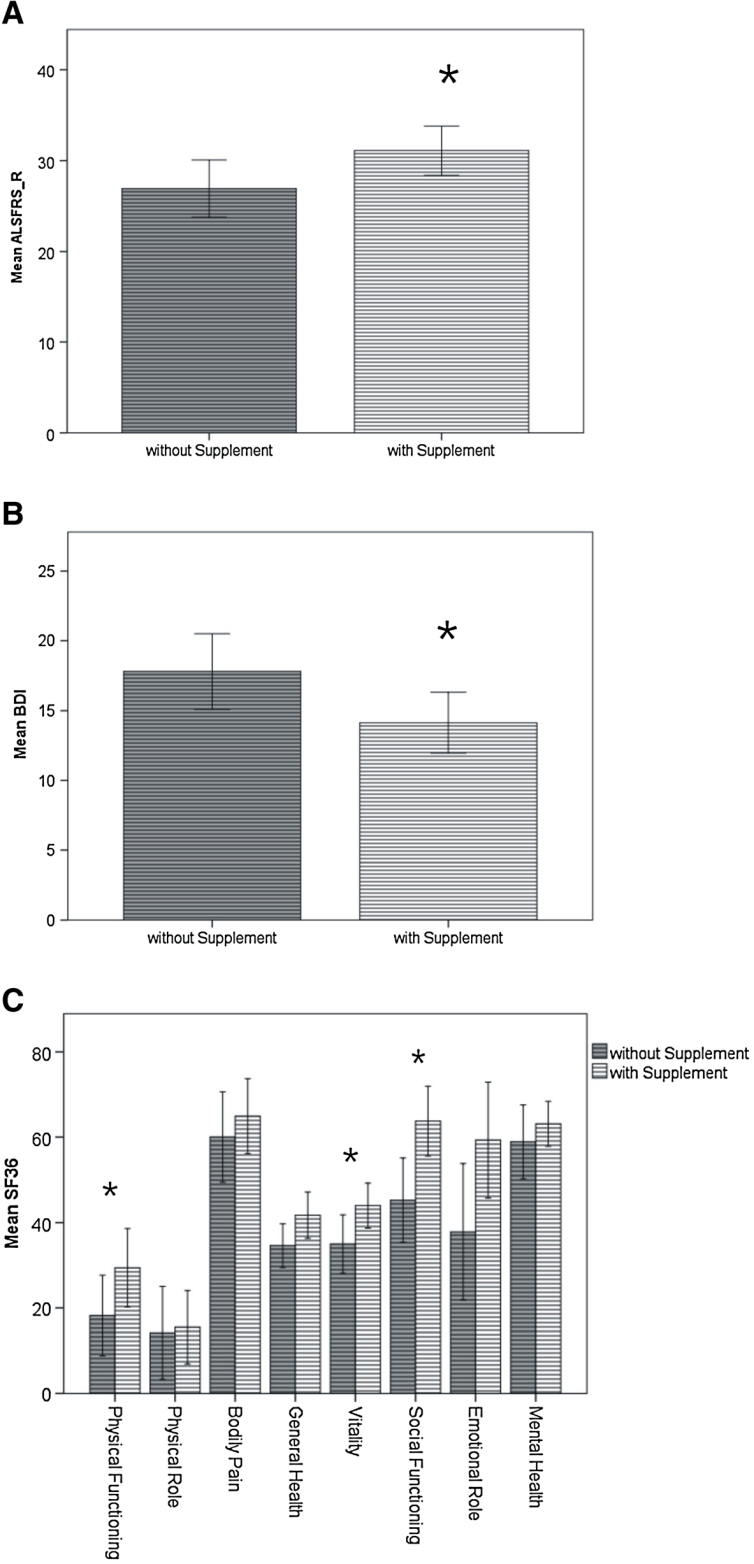
**Mean scores of ALSFRS-R, BDI and SF36 of patients with and without intake of dietary supplements.** Mean scores of ALSFRS-R (**A**), BDI (**B**) and SF36 (**C**) of patients with and without intake of other (not high calorie) dietary supplements. Supplement intake was associated with significantly higher scores at the ALSFRS_R and the SF36 subscales “physical functioning”, “vitality” and “social functioning” and significantly lower scores in BDI. * p < 0.05.

54.5% (n = 67) of the patients stated regular intake of other (not high-calorie) dietary supplements (e.g. vitamins). 44.8% (n = 30) of them consumed more than one supplement, some up to five simultaneously or preparations containing up to seven ingredients. Overall 23 different supplements were mentioned (Table 4). Analysis of the ALSFRS_R showed an inverse correlation between disease severity and supplement intake (i.e. patients taking dietary supplements were significantly less impaired than those who did not) (Figure 3A). There also was a significant difference in the BDI scores as well as the SF-36 subscales “physical functioning”, “vitality” and “social functioning” between the two groups: patients with supplement intake were significantly better regarding mood and QOL (Figure 3B and C). However, multiple regression analysis again showed that these differences are mainly attributable to the discrepancies in disease severity as assessed by the ALSFRS_R (=confounding factor). Only the difference in “social functioning” between patients with and without supplement intake remained highly significant, showing an independent influence of both supplement intake and ALSFRS_R (Table 3C). Hence patients using dietary supplements are feeling less affected in their “social functioning” which means contact or visit family members, friends and neighbours. However it could also be the other way around, meaning that patients with a more active social life are more likely to start taking dietary supplements. At the follow-up telephone interview two years after the first survey, 42.9% of the patients who had initially reported use of dietary supplements now declared that they had stopped any supplement intake.

**Table 4:** **Nutritional supplements and functional foods taken in our population**[15]

**Nutritional supplement**	**Mechanism of action (hypothesis)**	**Percentage (%) in our population**
Magnesium	Against cramps	63,6
Vitamin E	Antioxidant	40,3
Vitamin B (6 + 12)	Other agent	13,6
Vitamin C	Antioxidant	10,6
Vitamins, not specified	Antioxidant	7,6
Homeopathic medication		4,5
Folate	Other	4,5
Calcium	Bone regeneration	3,0
Carnitin	Antioxidant/Mitochondrial stabilizer	3,0
CoQ10	Antioxidant/Mitochondrial stabilizer	3,0
Schussler salts		3,0
L-Arginin	Muscle regeneration	3,0
Zinc	Other	3,0
Vitamin A	Antioxidant	1,5
Lipoic acid (Omega3)	Antioxidant/Anti-glutamate	1,5
Grape seed extract	Antioxidant/Anti-glutamate	1,5
Vitamin D	Other	1,5
Lycopin (tomato)	Antioxidant, Radical scavenger	1,5
Selen	Radical scavenger	1,5
Enzymes (Papain, (Chymo)Trypsin)	Antithrombotic, eupeptic	1,5
Protein preparation	Muscle regeneration	1,5
Willow capsules	Against pain	1,5
Chlorella	Against heavy metals	1,5
Himalaya salt		1,5
Not specified		12,1

## Discussion

According to the literature, weight loss is associated with shorter survival. In our ALS population it was accordingly connected with a significantly worse mean ALSFRS_R score and a significantly higher death rate after two years. Weight loss had a significant impact on QOL regarding the SF36 subitem “vitality”, meaning that it made patients feel more exhausted, tired and spiritless, regardless of the respective disease stage. Nevertheless, high calorie nutritional supplements were only consumed by one third of patients with weight loss, mainly due to the fact that the costs are often not covered by the respective health insurances. According to our survey, substantial benefit can be obtained from high calorie supplements and they should therefore be given more frequently. Only one quarter of patients with dysphagia had undergone PEG placement. Most of these patients reported weight stabilization and improvement of QOL. The decision about PEG insertion is mostly based on nutritional status and presence of dysphagia [16,17]. From clinical practise it is known, that patients are often cautious accepting a PEG, even when food intake becomes time consuming and laborious. Neurologists also tend to postpone the discussion of PEG [18]. As a result, PEG placement is often initiated too late and therefore associated with higher complication rates as it is known that FVC >50% at the time of PEG insertion is an important prognostic factor [16,18-20]. Our data confirm that PEG placement can stabilize body weight [10,21,22] and suggest additionally an impact on the QOL. The impact of PEG on QOL has been studied very little so far. Beside anecdotal impressions, only one study collected quantitative data: here, the majority of the patients mentioned stabilized nutritional and hydrational status as most positive effect of PEG. Fewer patients listed less fatigue or less time spent on meals and improved psychological wellbeing as positive effects of PEG [21,23]. Interestingly, in our cohort, even patients who had undergone further weight loss after PEG insertion, unanimously stated improvement of QOL. This suggests that other factors such as reduction of time consuming and tiring food and fluid intake with frequent choking are equally important for patients.

A further important result of our study is that weight loss *without* dysphagia is frequent in ALS. We aimed to define what distinguishes these patients from patients without weight loss. As the groups did not differ regarding site of onset and gender, these factors could not be relevant. While thorough neuropsychological testing had not been routinely performed, clinical signs of dementia were only detectable in one of the patients without weight loss, so that weight loss in the patients without dysphagia in our cohort could not be attributed to major cognitive or behavioural abnormalities. Patients with weight loss without dysphagia did not show differences in disease severity, grade of depression or presence of muscle fasciculations, but they did report higher respiratory efforts. It remains unclear how far this contributes to the weight loss. Two studies in ALS patients did not detect a relation between forced vital capacity (FVC) and energy expenditure (REE) [2,24] while two others suggest that increased respiratory efforts cause elevated REE [25,26]. Based on the existing literature, this increased energy consumption in ALS can probably not only be explained by respiratory insufficiency but rather by general hypermetabolism which occurs in about 50% of ALS patients and whose origin has not yet been fully elucidated [4,24].

The proportion of patients taking other, not high calorie dietary supplements such as vitamins was lower in our cohort (with 54.5%) than in the literature, were the percentage of supplement intake among ALS-patients is estimated as approximately 80% [7,10].

The observed differences in disease severity, depression and QOL are most probably not a direct effect of these diverse dietary supplements. While one must take into account a certain placebo-effect, the most likely explanation is that patients self-medicating with dietary supplements presumably represent a more hopeful and optimistic subgroup. This assumption is supported by the fact that they had higher SF36 “social functioning” scores, i.e. felt less affected in their interactions with family members, friends and neighbours. A more active social life may also have provided increased stimulations to try alternative treatment approaches. Discontinuation of dietary supplements over time (as revealed by our two-year follow-up interview) presumably is the result of loss of hope generally associated with further disease progression.

Although there is a lack of evidence for any relevant benefit of dietary supplements, as long as there is no clear contraindication and as long as efficient neuroprotective drugs beyond riluzole have not been discovered, self-medication with dietary supplements may represent hope and confidence for some patients and thereby have a positive impact on the disease course and quality of life.

## Conclusion

The significance of the present study is limited because it is retrospective and based on subjective data from the patients themselves. Nevertheless it provides valuable information that can be used as a starting point for further prospective investigations.

Even though malnutrition is a significant and independent prognostic factor in survival, it is often inadequately addressed in clinical practice. According to our results the effect of high calorie nutritional supplements and PEG is often higher than expected. Patients, caregivers and physicians should therefore be encouraged to consider these measures. However, their benefit still requires further confirmation by prospective studies.

To evaluate the reasons of weight loss beside dysphagia, further prospective analysis of patients without weight loss in comparison to patients suffering from weight loss not attributable to dysphagia, comparing clinical parameters such as fasciculations, spasticity and cognitive or behavioural abnormalities together with REE and FVC will certainly provide more thorough understanding of this phenomenon. In any case the existence of these different phenotypes highlights once more the heterogeneity in the clinical presentation of ALS.

Regarding dietary supplements, further studies are needed to evaluate the safety and efficacy of numerous dietary supplements and to enable appropriate recommendations.

## Abbreviations

ALS: Amyotrophic lateral sclerosis; ALSFRS_R ALS: Functional rating scale – revised version; BDI: Beck depression inventory; BMI: Body mass index; FVC: Flow vital capacity; PEG: Percutaneous endoscopic gastrostomy; QOL: Quality of life; REE: Resting energy expenditure; SF36: Short Form 36 health survey.

## Competing interest

The study was not externally funded. The authors declare no conflict of interest in regarding this manuscript.

## Authors’ contributions

SK and SP designed the study and drafted the article. MH and KK contributed to acquisition of data and revised the article critically for important intellectual content, SK and AZ analyzed and interpreted the data, AZ also revised the article critically for important intellectual content. RD and VS contributed to conception of the study and revised the article critically for important intellectual content. All authors give final approval of the version to be published.

## Pre-publication history

The pre-publication history for this paper can be accessed here:

http://www.biomedcentral.com/1471-2377/13/84/prepub
